# Promising Recent Strategies with Potential Clinical Translational Value to Combat Antibacterial Resistant Surge

**DOI:** 10.3390/medicines6010021

**Published:** 2019-01-31

**Authors:** Partha Karmakar, Vishwanath Gaitonde

**Affiliations:** 1 Department of Radiology, Washington University School of Medicine, St. Louis, MO 63110, USA; 2 Cambrex High Point, Inc., High Point, NC 27265, USA

**Keywords:** MDR surge for bacteria, antibiotic nanoparticles, targeted drug delivery, in vivo efficacy, clinical translation

## Abstract

Multiple drug resistance (MDR) for the treatment of bacterial infection has been a significant challenge since the beginning of the 21st century. Many of the small molecule-based antibiotic treatments have failed on numerous occasions due to a surge in MDR, which has claimed millions of lives worldwide. Small particles (SPs) consisting of metal, polymer or carbon nanoparticles (NPs) of different sizes, shapes and forms have shown considerable antibacterial effect over the past two decades. Unlike the classical small-molecule antibiotics, the small particles are less exposed so far to the bacteria to trigger a resistance mechanism, and hence have higher chances of fighting the challenge of the MDR process. Until recently, there has been limited progress of clinical treatments using NPs, despite ample reports of in vitro antibacterial efficacy. In this review, we discuss some recent and unconventional strategies that have explored the antibacterial efficacy of these small particles, alone and in combination with classical small molecules in vivo, and demonstrate possibilities that are favorable for clinical translations in near future.

## 1. Introduction

Sir Alexander Fleming famously quoted “Nature makes penicillin, I just found it” on his discovery of penicillin in 1929 [1]. Since their inception in the early 20th century, antibiotics have revolutionized the healthcare system. The advent and use of antibiotics has contributed immensely in the fight against pathogenic bacterial infection, saving millions of lives, and as such has transformed the human condition. In short, antibiotics were treated as a “Wonder Drug”. Tragically, this is no longer the case, and in the current global plight, the surge of antibacterial resistance has forced us into the post-antibiotic era [2,3]. The latest WHO Global Antimicrobial Surveillance System (GLASS) reports antibiotic resistance cases in half a million individuals across 22 countries [4].

Antibiotics are broadly classified as either bactericidal or bacteriostatic, depending on their ability to either kill or arrest growth of the bacteria. The majority of antibiotic classes target the disruption of bacterial cell wall synthesis via a complex mechanism that ultimately leads to cell death. The process may also inhibit the synthesis of protein, DNA and/or RNA, and usually exerts its effects on multiple targets [5,6,7]. Alternatively, the antibiotics may inhibit the protein synthesis at the ribosomal sites through their passage into the bacterial cell wall [8]. Targeting the ribosomal complex results in abnormal protein synthesis, generating lethal effects [9,10]. Antibiotics can also exert their action through interference of metabolic reactions. 

**Figure 1:**
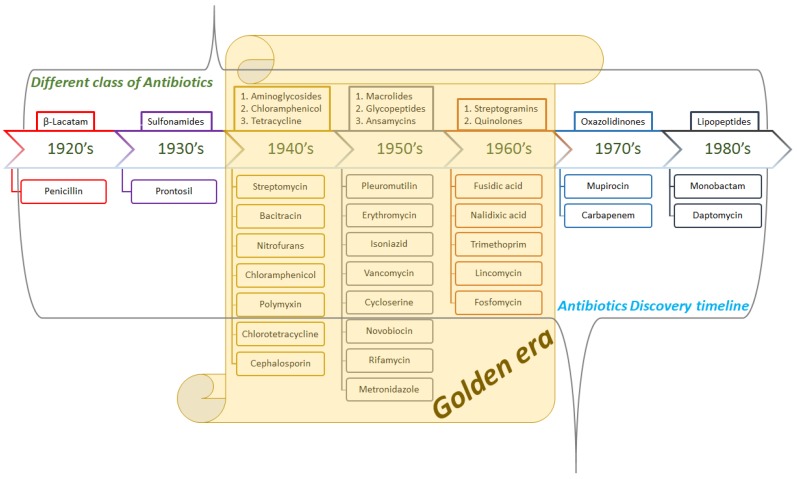
Schematic representation of the brief history of antibiotic treatments leading up to the surge in antibiotic resistance.

The occurrence of antibiotic resistance was registered prior to its introduction in the treatment of the bacterial infection [11]. Major classes of novel broad- and narrow-spectrum antibiotics were discovered between 1940 and 1960 in what came to be known as antibiotics, termed the “Golden era” (Figure 1) of antibiotic discovery. Treatments that were discovered in that timeframe, including Aminoglycoside, β-Lactam, Glycopeptides, Lipopeptides, Macrolides, Oxazolidones, Quinolones, Ansamycin, Streptogramins, Sulfonamides, and Tetracycline, have developed multidrug resistance (MDR) against bacterial pathogens. The MDR event eventually gave rise to bacterial strains that are now described as superbugs [12]. During the MDR snag, the superbugs thrived primarily due to their advanced ability to undermine antibiotic efficacy through genetic resistance and mutation [13,14,15]. 

Insights into the mechanism of antibiotic resistance suggest that the reason lies in the genetic, biochemical and physiological integrity of the pathogenic bacteria [16]. Genetic resistance is developed by the acquisition of DNA through gene transfer via either pilus-mediated fusion or through the process of transduction. Resistance induced by antibiotic alteration is observed where genetic mutation leads to the production of antibiotic deactivating enzymes. Additionally, genetic mutations may induce active-site receptor tampering targeted by the antibiotic treatment [17]. In gram-negative bacteria, the predominant form of resistance occurs through the efflux pump mechanism, which leads to limited bioavailability of the antibiotic. This mode of resistance results in poor antibiotic efficacy [18]. 

Even more alarming is the antibiotic resistance towards gram-negative bacteria. Urinary tract infection (UTI), spontaneous bacterial peritonitis (SBP) and bloodstream infection (BSI) are some of the most common and widespread health-related issues globally [19,20,21,22,23]. The challenge faced by researchers is gaining access the gram-negative bacterial cell, bypassing the hurdles via efflux pump and passage through the formidable double membrane permeability barrier. The framework of gram-negative bacteria, consisting of the outer membrane (OM), cytoplasmic membrane (CM), and a range of efflux pumps operating synergistically, reinforces its capability to debilitate antibiotic efficacy. A result of this synchronization is that the antibiotics implemented in the treatment of gram-negative bacterial infection either fail to cross over the OM (e.g., Polymyxin); CM (e.g., β-Lactams) or expelled by the efflux pump, leaving only a diminutive concentration of the antibiotic to which the bacterial machinery develops resistance, leading to MDR [24]. The aforementioned barriers to gram-negative treatments make designing antibiotics that act by evading the OM, CM and the efflux pump far more labyrinthine. As a result, the class of antibiotics that function against gram-negative bacteria have been limited. 

In the prevailing situation, bacteria have developed resistance to practically all classes of antibiotics. The antibiotic resistance emergency has consequences in the form of factors such as: 

(i) The current state of the pharmaceutical industry with respect to non-lucrative investments that ultimately leads to withdrawal or hesitance to enter the field [25,26]. The scale back is partially because economic returns associated with antibiotics are not perceived to be in the same financial tier as some other chronic disease conditions [27]. The landscape of antibacterial drug candidates has dramatically changed from novel and/or rational design to more of a “me too” style of candidate selection. This is particularly worrisome, as it is recognized that resistance to an antibiotic triggers resistance to antibiotics embodying that specific class. 

(ii) Unregulated dosing of antibiotics in humans, animals and agricultural activities aggravates the resistance, as it is reciprocal with intake [28,29]. This constitutes a sizable impact via the food-chain system, and also through animal discharge, which permeates into the environment and propagates antibiotic resistance [30,31]. 

The above factors incorporate the lack of innovation, and have strengthened the upwards trend in resistance; under these circumstances, it would be appropriate to anticipate that antibiotic resistance is inevitable for the foreseeable future. 

Brown, D. E. and Wright, G. D. mentioned that the narrow and outdated selection criteria for antibiotics are a major reason for the lack of innovative therapeutic strategies against MDR [2]. Hence, development of novel antibiotics alone cannot meet the present-day need, and invention of multiple new therapeutic strategies with broader and unbiased application is required to obtain effective therapeutic outcomes against the MDR. Ever since the new era of nanoparticles (NPs) began, their antibacterial applications have been reported. The potential for use in new therapeutic applications has been slow to gain momentum. A primary cause for this is the toxic effect of multiple 1st-generation metal nanoparticles against the eukaryotic cells. The hurdle to clinical translation is a well-known challenge [32,33,34,35,36]. Secondly, the clustering of the nanoparticles either in vitro or in vivo along with high liver opsonization has been a problem for clinical transformation [37,38,39]. Fortunately, towards the end of the present decade, nano-platforms are being explored in multiple ways to reach novel strategies to overcome the MDR [40]. In the present review, we discuss some of these strategies that have potential for clinical translation. 

It has been shown that varied sizes and shapes of the NPs play an important role in their antibacterial efficacy and mechanism of action [41,42,43,44,45,46]. In many of the strategies, typical NPs are transformed into films and other multidimensional complex structure to obtain the working platform in order to address the clinical translation challenges. We coin the term “small particles” (SPs) to describe all these different NPs and their respective application forms here in this review in contrast to the term “small molecules” (SMs), which is widely used for the classical antibiotic drugs.

## 2. Mechanism of Action of the SPs

**Figure 2:**
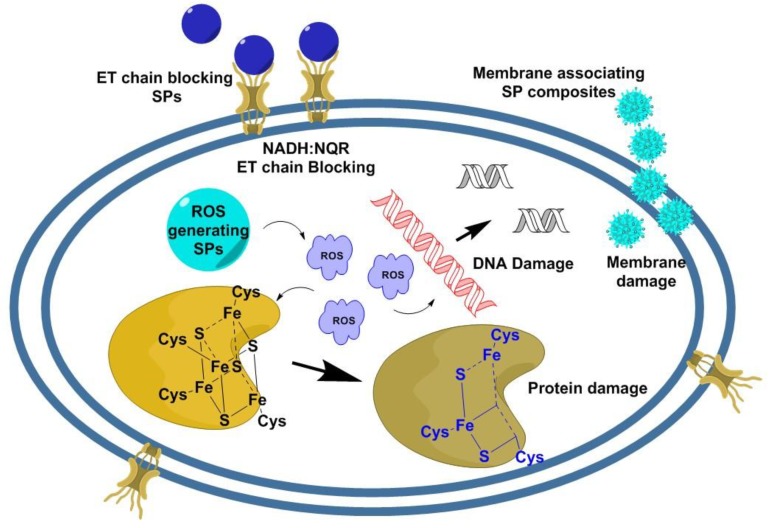
Mechanisms of action of different antibacterial nanocomposite platform (SPs). The various possible paths of bacterial cell killing by the SPs involve: (i) association of the SPs with the bacterial cell membrane via electrostatic or van der Waals interactions, leading to membrane damage; (ii) generation of ROS, leading to bacterial protein, membrane and DNA damage; and (iii) some metal-based SPs can release metal ions that block NADH: NQR electron transport chain [40,56].

The mechanism of action of different SPs against bacteria have still not been well understood, and more research is needed in this field [47,48,49]. Some of the widely accepted theories of mechanism involve generation of toxic Reactive Oxygen Species (ROS) initiated by the discharged metal ions from the metal NPs or the SPs [50]. The ROS can inhibit the bacterial growth by bacterial DNA damage via DNA bond dissociation and protein damage via disulfide bond dissociation at the active cite [51,52,53,54,55] (Figure 2). Perturbation of bacterial metabolic pathway, membrane surface charge disorder, mitochondrial disruption and internal pH modifications have also been reported as effective mechanisms of action of different SPs [40,50]. NADH: quinone oxidoreductase (NQR) electron transport (ET) channel is an important respiratory chain that maintains redox-driven trans-membrane Na^+^ potential in bacterial cell environments [56,57]. Positively charged silver (Ag) NPs that are able to release Ag^+^ ion can inhibit bacterial function by disrupting the NADH: NQR ET channel [58,59]. The polar attraction between the negatively charged bacterial cell walls and the positively charged SP surface, receptor-ligand binding, van der Waals interactions and several hydrophobic interactions between the bacterial cell wall and various different kinds of SPs leading to bacterial surface disruption are also attributed as the cause of antibacterial effects of these SPs [60,61]. 

In this context, it is worth mentioning that the association mechanisms of different SPs to gram-negative and gram-positive bacteria are different in many cases. As a result, SPs can be more effective toward a particular class of bacteria [62]. 

There are two main areas of antibacterial research using SPs. One is as a tool for efficient drug-delivery platform for the “small molecules” antibiotics. The other is the use of the SPs themselves as potential antibacterial agents. 

## 3. Small Particles as Delivery Vehicles for Small Molecule Antibiotics

At the initial level of antibiotic resistance, the conventional means to overcome the ineffectiveness was either to administer high dosages of the antibiotics [63,64], or to use combination antibiotic administration [65,66]. Unfortunately, these strategies resulted in high off-target toxicity and more drug resistance in the long run [67]. Nanoparticles have been in use for efficient drug delivery for a while, and some nanoparticle formulations are in clinical use or in various stages of clinical trials [24,68,69,70,71,72,73]. The high success rate of NPs as drug carriers is due to three main reasons: controllable size; selectivity, with less off-target toxicity; and drug-release controllability. These advantages have long been recognized for antibacterial drug delivery as well [74]. Until recently, there have been very few examples, but the present decade shows a robust strategy that can utilize all the benefits of the NP-delivery system for effective antibiotic delivery in vivo with potential for clinical translation. For example, gold nanoparticles have been successfully coated with ampicillin, kanamycin and streptomycin, and showed improved cytotoxicity [50,75,76]. Unfortunately, these studies are limited to in vitro efficacy comparison of the nano-composites SPs, and does not provide any further insight into future translations. Recent NP-based drug delivery strategies have shown promise for future clinical translation and are discussed in the sections below.

### 3.1. Bio-Mimetic and Bio-Compatible SPs for Targeted Antibacterial Drug Delivery Strategies

Bio-mimetic small molecules and SPs always have a high propensity for target accuracy, as well as faster clinical translation [77,78,79,80]. A natural cell membrane coating on synthetic NPs augments their therapeutic effect by triggering natural cell functions [81,82,83,84]. *H. pylori* is one of the major causes for peptic ulcer, gastritis and gastric cancer, affecting a large population worldwide [85,86,87,88]. *H. pylori* is known to bind strongly to the gastric epithelial cells through multiple mechanisms [89,90,91,92,93,94]. Angsantikul et al. recently adopted the cell membrane coating strategy to develop a Clarithromycin (CLR)-loaded gastric epithelial AGS cell-coated poly(lactic-co-glycolic acid) (PLGA) polymeric cross-linked nanocomposite small particle to target *H. pylori*. This approach leverages its strong affinity to AGS cells [95]. A superior antibacterial effect was demonstrated both in vitro and in mice models, along with specific binding of the nanocomposite SPs to the *H. pylori* bacteria [95]. Moreover, the oral delivery method optimized for the CLR loaded AGS-NP composite makes the administration more relevant for clinical translation. Mutation has already provided a bacterial strain resistant to CLR, and other antibiotics, including metronidazole, amoxicillin and levofloxacin, have also been ineffective recently [96,97,98]. Therefore, development of a new strategy is essential at this point to treat this infection. The strategy described by Angsantikul et al. has high potential to delay the antibacterial resistance to the recently used drugs against *H. pylori*, at least until a more robust method is developed.

Due to the surge of antibacterial resistance, there have been only limited options for the treatment of the superbug Methicillin-resistant S. *aureus* (MRSA) [99]. A horizontal transfer of the *van* gene cluster from *Enterococcus faecalis* (VRE) to MRSA introduces a high level of resistance to *S. aureus* [100,101,102], resulting in a 1000-fold reduction in vancomycin binding affinity to the bacterial surface ligand [103,104]. 

SPs containing magnetic nanoparticles have been explored as effective targeted antibiotic carriers, as well as bacterial detection and purification tools, since they can be maneuvered to bind to the bacterial surface [105,106,107]. Recently, commercially available high paramagnetic nanoparticles have been coated with human serum albumin (HSA) and the surface of the coated NPs were functionalized with vancomycin [108]. The nanocomposite reduced the minimum inhibitory concentration (MIC) values to 13–28 µg/mL compared to 250–4000 µg/mL of the free drug [108]. The increased binding affinity of the nanocomposite resulted bacterial membrane damage in two hours compared to the free drug, which was completely ineffective after 10 hours [108]. Though the present work only investigated the in vitro efficacy, the nanocomposite has high translation potential. The nanocomposite contains clinically compatible HSA coating on the surface of the magnetic nanoparticle. HSA is widely used in clinic; HSA-formulated highly toxic paclitaxel (Abraxane) is an FDA-approved chemotherapeutic drug [109]. Vancomycin was conjugated with the HSA through PEG linkers via click conjugation, which can enable the nanocomposite to be chemically stable for a longer period [108]. The HSA layer and the PEG linker can be helpful in enhancing the blood circulation time of the nanocomposite SPs in vivo [110,111,112]. It is worth mentioning here that biocompatible iron oxide-based SPs, which are well known for exerting magnetic properties, have recently been investigated widely as effective antibiotic carriers in in vitro systems and showed potential for further in vivo studies [113,114,115,116].

### 3.2. Bio-Mimetic and Bio-Compatible SPs for Immune System-Targeted Antibacterial Drug Delivery Strategies 

At the time of bacterial infection, phagocytic cells, specifically macrophages, perform killing of infected cells in the body [117,118,119]. Unfortunately, in many cases, some bacteria survive inside the macrophages, thus evading the immune system and leading to recurrence when a suitable environment is found [120,121]. Hence, targeting macrophages to kill residual bacteria is a strategy that has been duly investigated [122,123,124]. The efficiency of macrophages as target-specific drug carriers has recently been explored, and they represent the potential for a wide variety antibacterial treatment strategies [125,126,127]. Macrophages are able to detect and respond to any endogenous stimuli generated from infection, injury and disease [128]. These sentinel cells of the immune system, which also act as antigen-presenting cells [119], form the first line of defense for any bacterial infection and recognize a diverse array of microbial molecular patterns, largely via the toll like receptors (TLRs) binding mechanism [129]. 

Drug-loaded liposomes for targeted delivery to the macrophages [130] and liposomal vaccine delivery augmenting TLR binding have been well explored for development of antibacterial and anti-cancer vaccines [131,132,133]. Rukavina et al. recently demonstrated the efficacy of azithromycin-loaded liposomes to treat topical MRSA infections [134]. The phospholipid bilayer with charge and zeta-potential tenability of liposomes is advantageous for bacterial membrane association. Moreover, some of the liposomal drug formulations are FDA approved and are in clinical use [135]. Therefore, this strategy has high potential for clinical translation. 

Macrophages have high mannose receptor expressions [127,136]. Xiong et al. recently utilized these advantages to develop a mannosyl ligand-conjugated PEG and polyphosphoester cross-linked nanogel for antibacterial drug delivery [137]. The nanogel forms a hydrophilic core that can efficiently encapsulate hydrophilic small molecule drugs [137,138]. Vancomycin resistance has recently become a serious concern in multiple bacterial treatments [102,139,140]. Xiong et al. demonstrated the efficacy of their therapeutic strategy by loading vancomycin in the nanogel as the model antibiotic [137] against the resistant MRSA. Once administered, the engineered mannosyl arm of vancomycin-loaded nanogel SP composite binds to the mannose receptors of the macrophages and results in receptor-mediated cell uptake. The nanogel-containing macrophages then reach the bacterial infection site and phagocytose the bacteria. The polyphosphoester core of the nanogel SP is then digested via phospholipase and phosphatase, which are produced in high concentrations due to bacterial metabolism and cell signaling pathways [137,138,141,142,143,144,145,146]. Thus, the nanogel SP releases a high dose of vancomycin for a superior inhibition of bacteria compared to free vancomycin. The in vivo efficacy of the nanogel SP was successfully demonstrated in MRSA-infected zebrafish embryo models. The nanogel SP treatment achieved 89% survival of the embryo compared to the 69% survival of the free vancomycin [137]. Though the strategy is demonstrated as a proof of concept with vancomycin as model, it is a broad platform for multiple drug delivery to the macrophages. 

### 3.3. Small Particle Composites with Externally Triggerable Drug Release Mechanisms

Controlled drug release at the infected area in vivo is always desirable for most effective therapeutic response with minimal non-specific toxicity. To overcome the hurdles of antibacterial resistance, a combination of multiple strategies has been suggested by Brown D. et al. [2]. Hydrogels have recently been shown to be a highly effective scaffold for small molecule antibacterial drug delivery platforms due to their long focal area retention time and low off-target diffusion [147,148,149,150,151]. Glycol chitosan (GC) hydrogels are water-containing hydrophilic three-dimensional polymer networks that have extensive applications, including cell culture and tissue engineering [152,153,154]. Polydopamine (PDA) NPs have been popular due to the synthetic leverage of producing them in controllable size and multiple biomedical applications [155,156,157,158]. Near-infrared (NIR) light has been widely used due to its deep tissue penetrability and accurate non-invasive manipulations and control [159,160,161]. Interestingly, PDA NPs can be loaded with various small molecule drugs that can be released on demand by NIR irradiation [162,163,164]. Gao et al. recently combined all these strategies to develop an injectable polydopamine (PDA)-ciprofloxacin (Cip)- glycol chitosan (GC) hydrogel small particle composite as a specific drug release platform under NIR light activation [165]. The PDA-Cip-GC hydrogel small particle composite (Gel-Cip) was able to inactivate ~99% *S. aureus* when irradiated with 808 nm NIR laser [165]. The combination strategy was also able to heal the *S. aureus*-mediated wound up to 93.6% within 4 days in a mouse model, while the other controls could achieve only 13.6%–52.7% healing efficacy [165]. The wound healing in the combination treatment was evident with uniform tissue arrangement, blood vessel and hair follicle formation, thickened epidermis and well-proliferated fibroblast formation [165]. NIR spectroscopy has been widely explored for clinical application [166]. PDA and hydrogels are well known bio-compatible materials, and they have the advantage of bio-degradability within a reasonable time. Thus, this strategy demonstrates high potential for fast clinical translation.

### 3.4. Bio-Mimetic and Bio-Compatible SPs for Immune System-Targeted Antibacterial Drug Delivery Strategies with Externally Triggerable Drug Release Mechanisms

As developing a more robust design is desirable to combat the antibacterial resistance surge, a combination of all of the strategies discussed above appears viable to deliver a highly effective treatment program. To demonstrate this idea, Wang et al. recently developed novel NIR activatable pretreated macrophage-membrane-coated gold-silver nanocage (GSNC) vehicles for small molecule antibiotic delivery [167]. Prior to coating on the nanocage, the macrophages were pre-treated with bacteria *Staphylococcus aureus* and *Escherichia coli* as model gram-positive and gram-negative bacteria in order to enhance the TLR2 and TLR4 expression on the macrophage membrane surface, which is responsible for gram-positive and gram-negative microbial recognition, respectively [129,167,168,169]. The GSNCs contain cavities that can be loaded with small molecule antibiotics. The antibiotics can be released “on demand” by NIR irradiation [170,171]. Along with the high targeting ability and triggerable drug release advantage, the significant in vitro and in vivo efficacy of the platform [167] enhances the chances of this strategy being a potential treatment plan in the clinics of the near future. 

## 4. Small Particles as Antibiotics

The antimicrobial activities of various different types of NPs, especially metal NPs, have been extensively reported over the last two decades. Unfortunately, most of the reports are limited to the chemical characterizations of the core NPs or their final SP form, and corresponding in vitro efficacy studies. Though many of those findings were highly promising for clinical translation, only a few of them have been further investigated in order to move forward with in vivo applications [47]. One possible reason could be that these particles do not follow the guidelines of classical medicinal chemistry that have governed the antibiotic research methodology until now. Considering the timelines of the reports of applications of the SPs in in vivo models, it can be said that the urgency of developing unconventional strategies was felt only at the end of the 1st decade of the twenty-first century, when MDR against the classical small molecules reached an alarming stage [172,173]. Advancements of the antibacterial SPs the clinical translations have been reported in the field of biomedical engineering, and these include, but are not limited to, wound dressings, bone cements, dental materials, and coatings on artificial organs and implant devices [40]. 

### 4.1. Small Particle Applications in Wound Healing

Bacterial biofilm formation is a very common phenomenon in any epithelial wound and impedes the normal wound healing process [174]. Any wound healing material, such as a bandage or an ointment, is expected to have an antibacterial effect in order to prevent biofilm formation and anti-inflammatory properties, along with fibroblast and epithelial tissue proliferation supports, to allow smooth healing without chronic infection [175]. The use of conventional antibiotics in wound dressings is facing serious challenges due to MDR, especially because MRSA is closely associated with most wound-related biofilm formation, causing an increase in mortality and morbidity rate [176,177]. Use of unconventional antibiotics, including SPs, has resulted in considerable progress in overcoming the challenge [178,179]. 

Due to the long history of antibacterial efficacy of AgNPs [47], many Ag-based small particles have recently been translated into clinical applications, and multiple other candidates are currently in the pipeline [178]. Fucidin, Tegaderm, Acticoat, Bactigrass, and PolyMem Silver are some of the FDA-approved Ag small particle-containing wound dressing materials that are commercially available [180]. A Ag/AgCl/Graphene oxide SP composite wound dressing system has recently been developed by Zhou et al. that has successfully healed 2nd-degree burns within 10 days. Zhou’s composite material demonstrated high antibacterial efficacy in mice model [181]. The stable and uniform character of this SP film, along with significant antibacterial effect against *S. aureus* with no eukaryotic cell toxicity, makes this method a potential candidate for clinical translation. 

An in situ gelatin-reduced porous AgNP/Chitosan SP film-containing wound dressing has recently been reported to be highly effective in successfully healing an implanted and *S. aureus*-infected wound within 15 days in a rabbit model [182]. Chitosan and gelatin are widely used in the food and clinical industries as antibacterial agents, food preservatives and thickening agents [183,184]. Moreover, chitosan has also been used in synthesis of different SPs that showed considerable in vitro antibacterial efficacy [185,186,187]. Hence, this strategy provides an advantage for this SP system in terms of rapid clinical translation. Mesoporous silica-based SPs have opened multiple avenues of medical application, especially as DNA and RNA carriers. This particular application is due to its high drug-carrying capacity and low in vivo toxicity [188]. Wang et al. recently developed a mesoporous silica-based SP that demonstrated rapid hemostasis and antibacterial activity in mice model [189]. 

### 4.2. Small Particles in Artificial Implants

Small particles have recently shown promise in multiple artificial implantation applications as an antibacterial modality. Titanium dioxide (TiO_2_), silver, silicon, calcium and phosphorous SPs have been explored as potent antibacterial coatings on medical implant devices like cardiovascular apparatuses and catheters [190,191,192,193]. Copper oxide (CuO) and zinc oxide (ZnO) SPs have been studied for orthodontic treatments [194]. In cases of maxillofacial prostheses, infrared- photoactivatable TiO_2_ SPs have been suggested for potential clinical applications due to their efficacy against MRSA [195]. A ZnO-cyclodextrin-cefepime SP prepared by Matrix Assisted Pulse Laser Evaporation (MAPLE) demonstrated good inhibition against bacterial biofilm formation at up to 24 h, with decent biocompatibility tested in mice model, and it has potential for further study to develop new coatings for artificial implants [196]. Polymethyl methacrylate (PMMA), commonly known as “bone cement”, is used as implant fixation in clinics for various orthopedic surgeries, and antibiotics are customarily mixed with it in order to obtain a slow release of the drugs in order to treat any post-surgery bacterial infection [197,198,199,200]. PMMA containing 1% 5–50 nm Ag SPs was reported to be more effective against MRSA EDCC 5246 clinical samples compared to Gentamicin-loaded bone cement [201]. Mesoporous silica particles and caprolactum-silica particles have been demonstrated to be effective for controlled antibiotic release in bone cements [202,203].

### 4.3. Small Particle–Small Molecule Dual Functional Antibiotics; the Ultimate Weapon

After considering all the examples discussed above, it can be envisioned that SP-SM dual antibiotics may appear significantly more effective against the MDR compared to a single component as effective antibiotic. Lee et al. designed an amoxicillin incorporating nano-diamond as a SP antibacterial combo that can be incorporated in Gutta Percha for root canal treatment [204]. Song Wang and co-workers recently devised an interesting strategy for diabetic wound healing using an antibacterial host-defense immunogenic peptide LL37 [205] engrafted in AuNPs of antibiotic efficacy [206]. Thus, the formed SPs showed highly synergistic antibacterial activity. The SPs were also demonstrated to be able to carry DNA, and both the diabetic wound healing efficacy and DNA transfection ability were successfully validated in mice model, demonstrating a high potential for translation.

Although progress is being made in combining established small molecule antibiotics with antibacterial small particles to achieve a synergistic effect, new small molecule drugs could enhance the possibility of success of this combination strategy by multiple folds. Though much slower than required, some small molecule antibiotics have shown promise against highly resistant bacteria [207]. N,N-dimethylebiguanide-metal complexes have been demonstrated to be an effective new series of metal-based small molecule antibiotics that is worth exploring for in vivo efficacy and toxicological studies [208,209,210].

**Figure 3:**
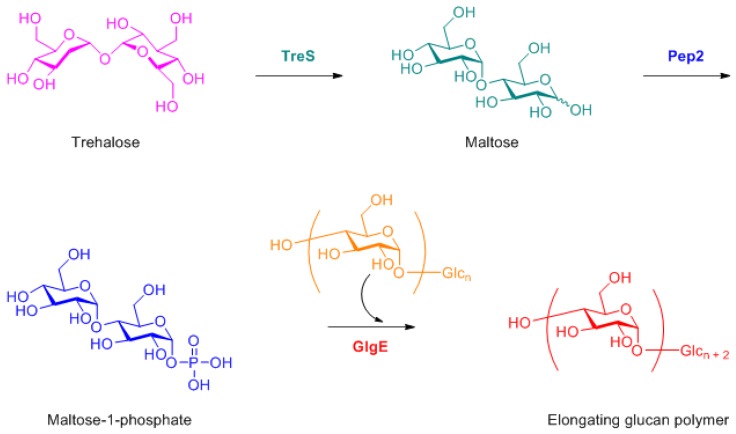
Biosynthetic pathway of α-1,4-glucan elongation via, TreS, Pep2 and GlgE [217-220].

The discussion about the MDR surge and the strategies to tackle the challenges cannot be completed without mentioning the present state of tuberculosis (TB). Tuberculosis has been a scourge among bacterial infections, listed as one of the world’s top ten causes of death, and the leading cause from a single infectious agent. The World Health Organization (WHO) reported an estimated 1.3 million deaths in 2017 as a result of TB [211]. Tuberculosis remains an unmet challenge in the medical and scientific community. One promising strategy is to target *Mycobacterium tuberculosis* GlgE (Mtb GlgE), a genetically validated tuberculosis target. GlgE (EC, 2.4.99.16) an α-1,4-glucan: phosphate maltosyltransferase is a member of the glycoside hydrolase family GH13_3 subfamily in the CAZy database [212,213,214,215], and it has been identified that GlgE (GlgE, Rv1327c) is vital for in vitro growth in Mtb [216]. The importance of GlgE arises from its involvement in the biosynthesis of the α-1,4-glucan 47 synthesis via transferring the donor maltose-1-phosphate (M1P) to the reducing end of the elongating α-1,4-glucan chain with an α-retaining mechanism (Figure 3). In recent years there have been sustained efforts to make a breakthrough in anti-tuberculosis drug development utilizing this key strategic information. Sucheck and co-workers developed a series of considerably effective trehalose and other glyco-conjugate-based Mtb GlgE inhibitors [217,218,219,220]. Another important chemical moiety from the classical medicinal chemistry viewpoint are the pyrazolo[1,5-a]pyrimidine and its derivatives. This core heterocyclic structure has been a consequential scaffold utilized in the design and development of some of the most promising antimicrobial compounds [221,222,223]. Along with the progress with small molecules, rapid progress is also underway in small particle developments against Mtb, where antibacterial efficacy is observed with immunotherapy [224]. 

## 5. Challenges and Prospects

As discussed, it appears that broader therapeutic strategies are being explored in order to leverage the maximum utility of SPs; however, there remain potential challenges that need to be overcome quickly. One major difference between classical small molecule antibiotic drug discovery and small particle drug discovery is that, unlike the small molecules, the mechanisms of action of small particles are not completely understood. Moreover, the field of SP antibiotic research is young and not well established. To obtain a fast outcome, there is an urgent need to develop a well-structured small particle-based drug discovery unit at an industrial scale that includes established steps of drug discovery research, including computational modeling, mechanistic study, small particle library synthesis, high-throughput screening, etc., along with unconventional combinatorial strategy development for multiple in vivo applications. 

## 6. Conclusions

In the surge of MDR, bacteria develop rapid resistance against almost all kinds of available small molecule antibiotics. New antibiotic and, just as importantly, new therapeutic strategies are required to meet the urgent demand of MDR. Combination therapeutic strategies involving small particles consisting of different forms of nanoparticles with antibacterial efficacy along with existing and new small molecule antibiotics have high potential to overcome this alarming era of MDR. In the present review, we have visited some of the recent innovations that have successfully demonstrated the effectiveness of these small particles, both as efficient cargo for small molecule antibiotic delivery and as effective antibiotics themselves. Contemporary research has shown promise for clinical translation. A well-organized effort and maturation of the field will prompt the industrial resources required to combat the current state of MDR.
